# Lympho-vascular invasion impacts the prognosis in breast-conserving surgery: a systematic review and meta-analysis

**DOI:** 10.1186/s12885-022-09193-0

**Published:** 2022-01-25

**Authors:** Yi-Ming Zhong, Fei Tong, Jun Shen

**Affiliations:** 1 Department of Medical Oncology, Sir Run Run Shaw Hospital, Medical School, Zhejiang University, No. 3 Road of Qingchun East, Zhejiang Province, Hangzhou, China; 2 The People’s Hospital of Longyou County, No. 373 Rongchang Road, Longyou County, Zhejiang Province, Quzhou, China; 3 Department of Surgical Oncology, Sir Run Run Shaw Hospital, Medical School, Zhejiang University, No. 3 Road of Qingchun East, Zhejiang Province, Hangzhou, China

**Keywords:** Breast cancer, Breast-conserving surgery, Lympho-vascular invasion, Prognosis

## Abstract

**Background:**

It is estimated that breast cancer (BC) incidence, especially that of early-stage breast cancer cases continues to rise due to increased universal screening. Breast-conserving surgery (BCS) is the main intervention for early-stage BC. Lympho-vascular invasion (LVI) is reported to influence breast cancer prognosis but its prognostic value in breast-conserving treatment is controversial.

**Methods:**

A search was conducted on the Cochrane library, PubMed, Web of Science, and EMBASE from inception to December 1^st^, 2021, without language restrictions, to identify studies that explored the prognosis of lympho-vascular invasion in breast-conserving surgery. Reviews of each study were conducted, and data extracted. The meta-analysis was performed with StataSE 16. Study quality assessment was evaluated using the Newcastle–Ottawa Scale.

**Results:**

Overall, 15 studies with 21,704 patients deemed eligible for this study. Event-free survival (EFS), disease-free survival (DFS), overall survival (OS), distant metastases (DM), loco-regional recurrence (LRR), local recurrence (LR), breast recurrence (BR), disease specific survival (DSS), and breast cancer specific survival (BCSS), were extracted from each study. We found that LVI leads to poor OS (HR = 1.46, 95% CI: 1.17–1.83), DM (HR = 2.08, 95% CI: 1.66–2.60) and LR (HR = 2.00, 95% CI: 1.54–2.61).

**Conclusions:**

We confirmed that early-stage BC patients with LVI-positive have poorer OS, DFS, LRR, BCSS, DM and LR following receiving BCS than those LVI-negative patients. Mastectomy, in combination with radical systemic therapies could be considered, especially in those requiring second surgery. How to change the impact of LVI on the local recurrence rate and long-term survival in patients who undergo BCS may be a valuable research direction in the future.

**Supplementary Information:**

The online version contains supplementary material available at 10.1186/s12885-022-09193-0.

## Background

Globally, Breast cancer (BC) accounted for about 2.26 million cases in 2020, surpassing the number of lung cancers [1–3]. Moreover, BC is the 5th most common cause of cancer-related deaths [1].

The proportion of early-stage breast cancers continues to rise due to universal screening [4]. Advances in chemotherapy, targeted therapy, endocrine therapy, and immunotherapy have greatly improved breast cancer survival [5–7]. Due to early diagnosis and better prognosis, breast-conserving surgery is often recommended. Although there were some concerns about the surgery at the beginning [8], the exploration and improvement of the surgical style has not stopped since 1970s [9, 10]. In 1996, sentinel lymph node biopsy (SLNB) was adopted in BC staging and treatment and has promoted the development of breast-conserving surgery (BCS, or known as breast-conserving therapy) for BC [11, 12]. For early-stage BC, breast-conserving therapy extends disease-specific survival relative to mastectomy [13] but a high local recurrence risk (15-year relapse rate of 15.9-21.4%) has been observed [14].

Numerous breast cancer prognostic factors have been identified, including disease pathological stage, molecular subtype, lymph node invasion, lympho-vascular invasion, and histological grade [15–19]. Clinical studies show that lympho-vascular invasion correlates with breast cancer lymph node metastases and poor prognosis [15, 16]. A positive margin is associated with increased local recurrence (LR) in early-stage BC patients receiving BCS [20, 21]. Moreover, lympho-vascular invasion and extranodal tumor extension are risk indicators of breast cancer related lymphoedema [22, 23], which may affect the further treatment plan [24]. However, correlation between lympho-vascular invasion and LR or survival in breast cancer after breast-conserving therapy is controversial [25–28]. LVI is not systematically taken into account in decisions on breast cancer surgery (not mentioned in the National Comprehensive Cancer Network (NCCN) [29], Saint Gallen guidelines [30], or the European Society for Medical Oncology (ESMO) recommendations [31]). This meta-analysis of published data aimed to establish the prognostic significance of LVI in breast-conserving surgery.

## Methods

### Literature search and study selection

A systematic literature screening was done on Cochrane library, PubMed, Web of Science, and Embase, from inception to December 1^st^, 2021, including all prospective and retrospective investigations. The search terms used on PubMed were: ((breast conservative therapy) OR (breast-conserving surgery) OR (reserved mastectomy)) AND ((lymphovascular invasion) OR (lympho-vascular invasion) OR (lympho vascular invasion) OR (tumor thrombus) OR (carcinoma embolus)). Other search strategies are shown in *Supplementary Material “Search strategy”*. The searches confirmed with the Preferred Reporting Items For Systematic Reviews and Meta-Analyses (PRISMA) 2020 statement [32, 33]. PRISMA 2020 checklist is shown in *Supplementary Material “PRISMA Checklist”*. Inclusion criteria were: 1) The study contains lympho-vascular invasion data after breast-conserving surgery; 2) Study has sufficient information for 95% confidence interval (95% CI) and Hazard Ratio (HR) analyses of the outcomes; 3) The study performed multivariate analyses. Exclusion criteria were: 1) Use of non-standard treatment; 2) Lack of distinction between LVI-unknown and LVI-positive patients in the analysis; 3) The survival data weren’t compared within BCS patients, for example: BCS patients with mastectomy patients. Case reports, letters, commentary articles, and conference abstracts were excluded.

### Retrieval and quality assessment of data

Two independent researchers (YZ and FT) performed retrieval and quality assessment of data. The information extracted included number of patients included in studies, year of publication, first author’s name, study type, median follow-up months, breast cancer subtype, treatment type, and outcomes. The HR and 95% CIs were extracted from each study and classified by different outcomes. Prospective studies were assessed using Cochrane RoB 2.0 tool [34]. The Newcastle–Ottawa scale (NOS) was applied to analyze retrospective studies [35].

### Statistical analysis

Pooled HR with 95% CIs (95% CI) were determined for all extracted outcomes (OS, DFS, EFS, LR, LRR, DM, BR, BCSS and DSS). *I*^*2*^ test was used to assess statistical heterogeneity. *I*^*2*^ >56% indicated significant heterogeneity. *I*^*2*^ < 31% indicated homogeneity. *I*^*2*^ between 31% and 56% indicated mild heterogeneity [36]. The meta-analysis was done by applying the random effect model. Egger, Funnel plots, and Begg tests were utilized for the assessment of publication bias. *P ≤*0.05 (2-sided) indicated as statistical significance. All analyses were carried out with the Stata, version 16.0 (Stata Corporation, College Station. TX).

## Results

### Study selection and quality assessment

**Fig. 1:**
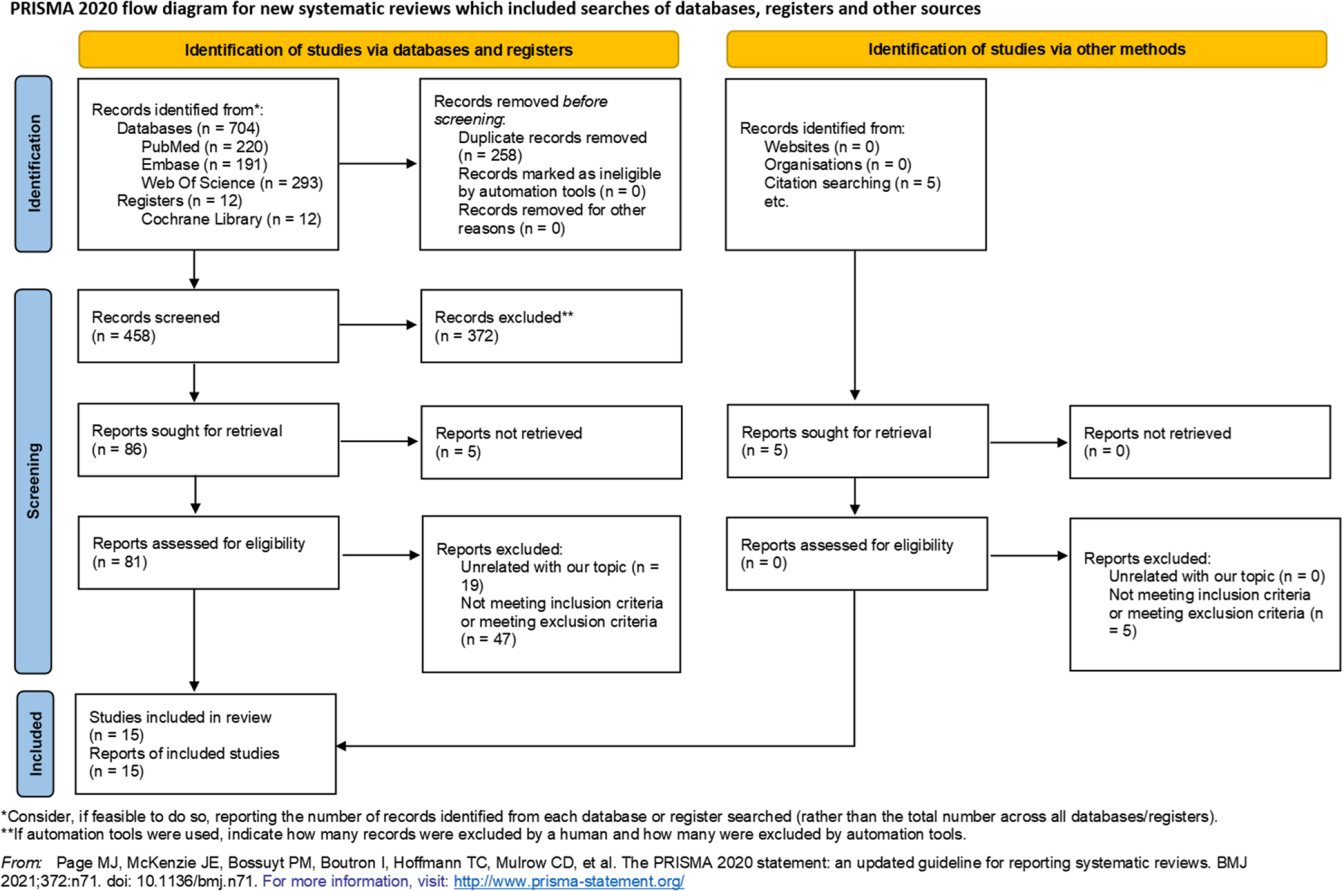
PRISMA 2020 flow diagram for meta-analysis

Our search strategy identified 716 records and 5 additional records were identified from references in these studies (Fig. 1). After 258 duplicate records removal, 458 records remained. Of these, 372 were excluded after title and abstract review. Of the remaining 86 records, 5 reports couldn’t be retrieved, 19 were excluded because of unrelated topic, 52 weren’t meeting inclusion criteria or meeting exclusion criteria, as shown in the flow diagram (Fig. 1). Finally, 15 full-text studies involving 21,704 patients were included this meta-analysis (Table 1). Among these, 3 were prospective and 12 were retrospective. The characteristics and quality of the studies are shown on Tables 1, 2, 3.

**Table 1:** The characteristic of each involved study

Study	Year	Country or Region	Study type	Number of patients	Median follow-up months	Subtype	Chemotherapy^a^	Radiotherapy^b^	Outcomes^c^
Magee B [37]	1996	United Kingdom	Prospective	708	96	Not mentioned	No	Mixed	BR (LR)
Dinshaw KA [25]	2005	India	Retrospective	1022	53	All	Adjuvant	Yes	OS/DFS/LRR/DM/LR
Yoshida T [38]	2009	Japan	Retrospective	2243	64.7	All	Adjuvant	Mixed	DM
Yi O.V. [39]	2009	Korea	Retrospective	578	54.1	All	Mixed	Yes	LR
Lupe K [40]	2011	Canada	Retrospective	2264	62.4	All	Not mentioned	Yes	LR
Adkins FC [41]	2011	America	Retrospective	1325	62	TNBC	Adjuvant	Mixed	LRR
Freedman GM [42]	2012	Brazil	Prospective	1478	68	All	Mixed	Yes	OS
Mittendorf EA [43]	2013	America	Prospective	2983	94.8	All	Mixed	Yes	LRR
Perez CA [44]	2013	America	Retrospective	704	51	TNBC	Adjuvant	Mixed	OS/LRR/DM
Matsuda N [45]	2014	Japan	Retrospective	622	51	All	Neoadjuvant	Yes	LRR
Park JS [46]	2015	Korea	Retrospective	1071	114	All	Adjuvant	Mixed	DFS/BCSS (DSS)
Sopik V[47]	2015	Canada	Retrospective	1675	157.2	All	Mixed	Mixed	BCSS (DSS)/LR
Nichol AM[26]	2017	Canada	Retrospective	1034	151.2	HR +	Not mentioned	Half patients received	OS/EFS
Lee BM[27]	2018	Korea	Retrospective	2206	73	All	Mixed	Yes	OS
Chen SY[28]	2018	China	Retrospective	1791	50.4	All	Adjuvant	Yes	OS/DFS/LRR/DM

*LR*, Local recurrence; *LRR*, Local–regional recurrence; *BR*, Breast recurrence; *DFS*, Disease-free survival; *BCSS*, Breast cancer-specific survival; *DSS*, Disease-specific survival; *DM*, Distant metastases; *OS*, Overall survival; *EFS*, Event-free survival

^a^ Chemotherapy is according to standard therapy, "Adjuvant" represents the study that excludes the patients who received neoadjuvant chemotherapy, "Neoadjuvant" means the study only includes the patients who received neoadjuvant chemotherapy, "Mixed" means the study contains all the BCS patients. Notice: "Adjuvant" and "Mixed" contain the patients who didn't receive the chemotherapy

^b^ "Yes" for the radiotherapy represents that the study excludes the patients who didn't receive the radiotherapy or received the nonstandard radiotherapy, "Mixed" means the study contains all the BCS patients

^c^ According to the original articles, BR is equal to LR, BCSS is equal to DSS. Univariate analysis has been excluded

**Table 2:** Cochrane RoB 2.0 tool evaluate for the RCTs

Study	Outcomes	D1	D2	D3	D4	D5	D6
Magee B et al. (1996) [37]	LR						
Freedman GM et al. (2012) [42]	LRR/OS						
Mittendorf EA et al. (2013) [43]	LRR						

Domains: D1: Randomization process

D2: Deviations from intended interventions

D3: Missing outcome data

D4: Measurement of the outcome

D5: Selection of the reported result

D6: Overall

Legend:

Low risk

Some concerns

High risk

**Table 3:** NOS scale for the cohort studies

Study	Selection	Comparability	Outcomes	NOS score
Dinshaw KA et al. (2005) [25]	3	2	2	7
Yoshida T et al. (2009) [38]	3	2	3	8
Yi O.V. et al. (2009) [39]	4	2	2	8
Lupe K et al. (2011) [40]	4	2	3	9
Adkins FC et al. (2011) [41]	3	2	3	8
Perez CA et al. (2013) [44]	3	2	2	7
cN et al. (2014) [45]	3	2	2	7
Park JS et al. (2015) [46]	3	2	3	8
Sopik V et al. (2015) [47]	4	2	3	9
Nichol AM et al. (2017) [26]	3	2	3	8
Lee BM et al. (2018) [27]	4	2	3	9
Chen SY et al. (2018) [28]	4	2	2	9

### Prognosis of lympho-vascular invasion after breast-conserving surgery

**Fig. 2:**
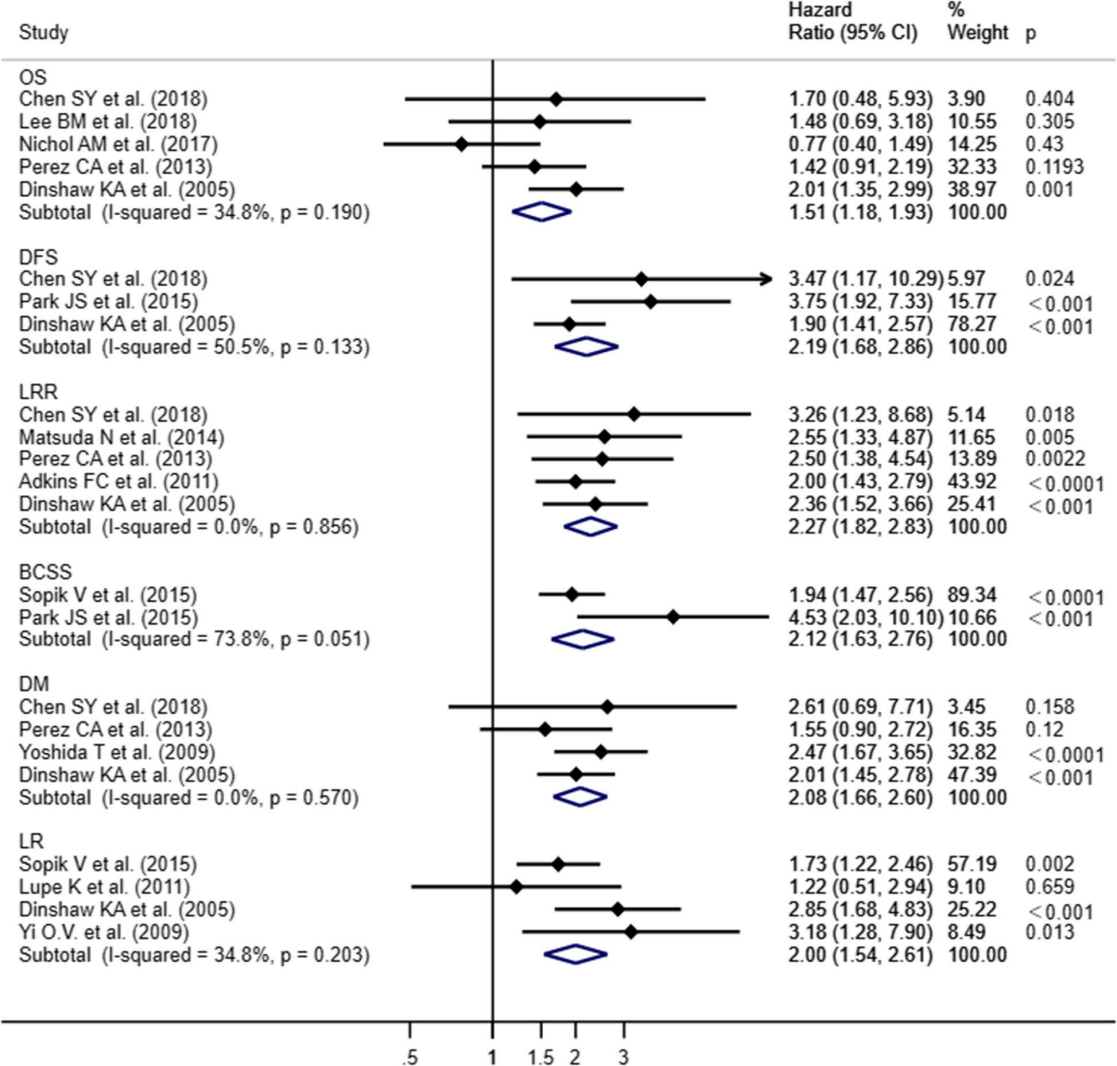
Forest plot of meta-analysis and cumulative meta-analysis in primary outcomes

Of the prospective studies, Freedman GM et al.[42] showed the outcomes of LRR and OS (HR = 1.254, 95% CI 0.944–1.667, p = 0.12), Mittendoff EA et al.[43] analyzed LRR (HR = 1.49, 95% CI 1.02–2.17, p = 0.039), and Magee B et al.[37] identified LR (HR = 1.78, 95% CI 1.035–3.063, p = 0.037) as outcomes. Additionally, the study of Freedman GM et al.[42] only contained HR for LRR, except for the 95% CI and *p*-value; Magee B et al. showed *e*^*β*^ (equal to Hazard Ratio), and *p*-value, in order to have the unification, we transferred these data into HR and 95% CI according to the functions conducted by Altman DG et al.[48] Because the three prospective studies involved different outcomes, the meta-analysis could not be applied on them. Thus, the meta-analysis was conducted on the retrospective studies assessed by random effects model. The HR and 95% CI data were separately pooled from each study. Outcomes included: overall survival (OS), disease-free survival (DFS), event-free survival (EFS), local recurrence (LR), loco-regional recurrence (LRR), distant metastases (DM), breast recurrence (BR), breast cancer specific survival (BCSS), and disease specific survival (DSS). According to the definitions in these studies, BR is equal to LR and BCSS is equal to DSS. Major outcomes in each study are shown on Table 1. The meta-analysis on retrospective studies showed that lympho-vascular invasion (LVI) after breast-conserving surgery significantly worsened OS, DFS, LRR, BCSS, DM and LR (Fig. 2). Results of heterogeneity tests are shown on Fig. 2. There were only two studies included in the BCSS, and the heterogeneity was relatively high (*I*^*2*^ = 73.8%, *p* = 0.051). On the other hand, the conclusion of each study both showed the significant difference in the BCSS outcomes, so we accepted this heterogeneity. Mild heterogeneity was also observed in DFS (*I*^*2*^= 50.5%, *p *= 0.133). Thus, we evaluated its similarity to that of BCSS and accepted the heterogeneity. Taken together, this meta-analysis found that LVI is a significant prognostic factor in early-stage breast cancer after BCS.

### Publication bias and sensitivity analyses

**Fig. 3:**
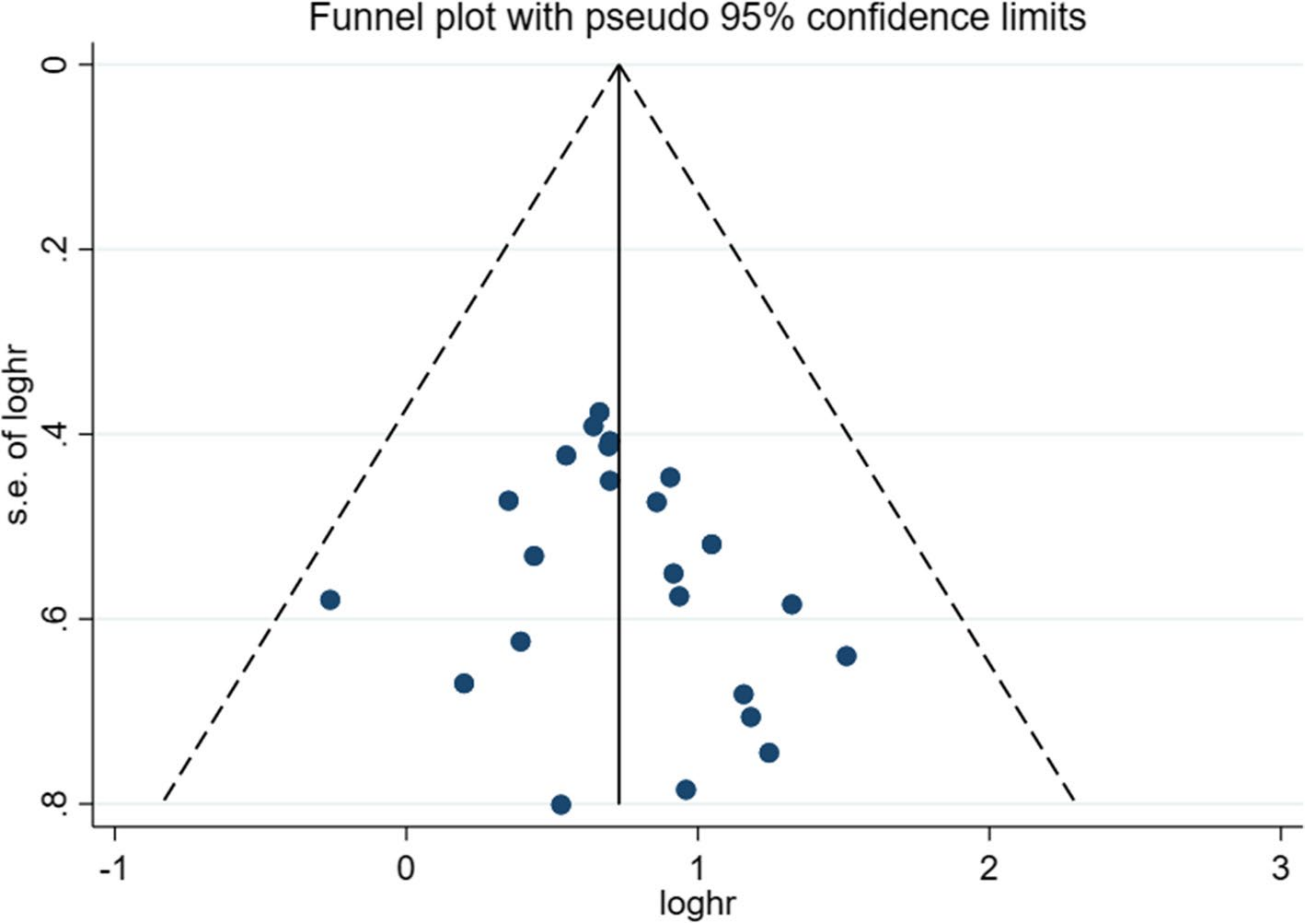
Publication Bias analysis for the meta-analysis

Funnel plots indicated a symmetric distribution of included studies. Begg and Egger tests (Begg = 0.112 > 0.05, Egger = 0.279 > 0.05) revealed no publication bias in these studies (Fig. 3), which was confirmed by sensitivity analyses.

## Discussion

Lympho-vascular invasion correlates with lymph node metastases and poor breast cancer prognosis [15, 16]. Breast-conserving surgery is a standard treatment option for early-stage breast cancer. However, few studies have focused on the prognostic role of LVI after breast-conserving surgery. This meta-analysis involved studies on breast-conserving surgery that contain LVI data. The prospective studies included showed the significant longer LR and LRR in LVI positive patients than LVI negative patients who underwent breast-conserving therapy, whereas with the similar OS [37, 42, 43]. However, we could not meta-analyze the prospective studies due to insufficient data. The conclusions of the retrospective studies included in our meta-analysis were controversial. DFS, LRR and BCSS were significantly different between LVI-positive and negative patients, studies showed a poor prognosis in LVI-positive patients; however, controversial conclusions were conducted in OS, DM and LR. In this meta-analysis, we conclude that early-stage breast cancer patients after breast-conserving surgery with LVI showed poorer OS, DFS, LRR, BCSS, DM and LR than those without LVI.

**Fig. 4:**
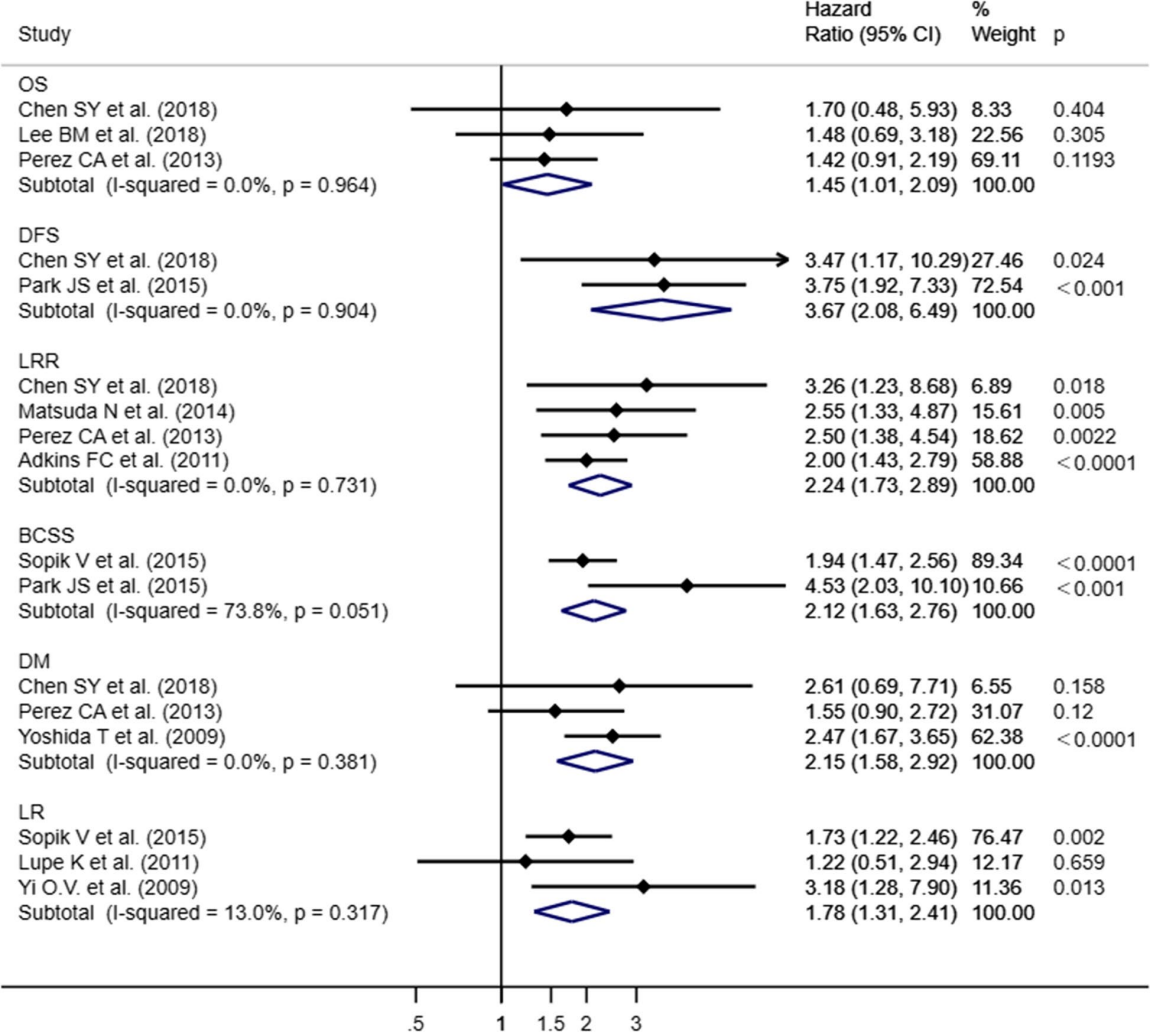
Forest blot of survival data for sensitivity analyses

We noticed that some studies chose OS as outcome, showed different trends in HR or *p*-value: In the study of Nichol AM et al. [26] the HR is less than 1 (HR = 0.77) with a *p*-value greater than 0.05 (*p* = 0.43), while Dinshaw KA et al. [25, 49] showed the significance in the OS (*p* = 0.001) and the HR is greater than 1 (HR = 2.01) (Fig. 2). Sensitivity analyses after exclusion of both studies and redoing the meta-analysis with the remaining studies also revealed significant difference (Fig. 4), and low heterogeneity (*I*^*2*^ = 0%, *p* = 0.954). Indicating that all these studies should be included in the meta-analysis.

A variety of patient, treatment, and pathologic factors have been reported to be associated with increased risk of local recurrence after breast conservation therapy. For breast cancer, positive microscopic margins are associated with a ≥ twofold higher risk of local recurrence relative to negative margins [20]. Thus, re-excision to achieve negative margins should be done for most patients with positive margins [50]. Our findings show that LVI positive breast cancer patients undergoing breast conservation therapy have a twofold higher risk of local recurrence relative to LVI-negative patients. Local recurrence after breast-conserving surgery for invasive cancer can influence patient survival. The Early Breast Cancer Trialists Collaborative Group (EBCTCG) found that 1 life is saved at 15-year follow-up for every 4 local recurrences prevented at 10 years after lumpectomy [51]. Our results show poor survival in LVI-positive breast cancer patients with breast conservation therapy. Thus, patients undergoing breast conservation therapy need to know about the predictive value of LVI on local recurrence and survival and mastectomy, with or without breast reconstruction should be considered, especially for patients who need positive margin re-excision. Recent studies show that breast reconstruction after a mastectomy has similar results to breast-conserving surgery in terms of quality of life [52].

Our meta-analysis based on retrospective studies may carry bias due to retrospective data analysis. The advent of molecular subtyping of breast cancer has changed the paradigm for breast cancer treatment. Neoadjuvant therapy has been standard care for human epidermal growth factor receptor 2 overexpressing, and triple negative breast cancers [53]. LVI in patients treated with neoadjuvant chemotherapy was an independent predictor of local recurrence, distant metastasis, and overall survival in all breast carcinomas [54]. Clinical studies on if LVI is an independent prognostic factor in stage T1N0M0 breast cancer, and if further systemic treatment or mastectomy can improve the prognosis of LVI-positive patients LVI are needed.

## Conclusions

We find that early-stage breast cancer patients after breast-conserving surgery with LVI showed poorer OS, DFS, LRR, BCSS, DM and LR than those without LVI. Mastectomy or its combination with radical systemic therapies could be considered, especially for patients who need a second surgery. How to change the impact of LVI on the local recurrence rate and long-term survival in patients who undergo breast-conserving surgery may be a valuable research direction in the future.

## Supplementary Information

**Additional file 1. **

**Additional file 2. **

## Data Availability

All data generated or analysed during this study are included in this published article and its supplementary information files.

## Abbreviations

OS: Overall survival
DFS: Disease-free survival
EFS: Event-free survival
LR: Local recurrence
LRR: Loco-regional recurrence
DM: Distant metastases
BR: Breast recurrence
BCSS: Breast cancer specific survival
DSS: Disease specific survival
LVI: Lympho-vascular invasion
BC: Breast cancer
BCS: Breast-conserving surgery
SLNB: Sentinel lymph node biopsy
NCCN: National Comprehensive Cancer Network
ESMO: European Society for Medical Oncology
PRISMA: Preferred Reporting Items For Systematic Reviews and Meta-Analyses
CI: Confidence interval
HR: Hazard Ratio
NOS: The Newcastle-Ottawa scale
EBCTCG: The Early Breast Cancer Trialists Collaborative Group

## References

[1] Ferlay J EM, Lam F, Colombet M, Mery L, Piñeros M, et al. Global Cancer Observatory: Cancer Today. Lyon: International Agency for Research on Cancer 2020 [updated April 2021; cited 2021 April]. Available from: https://gco.iarc.fr/today

[2] DeSantis CE, Ma J, Gaudet MM, Newman LA, Miller KD, Goding Sauer A (2019). Breast cancer statistics, 2019. CA Cancer J Clin.

[3] Sung H, Ferlay J, Siegel RL, Laversanne M, Soerjomataram I, Jemal A (2021). Global cancer statistics 2020: GLOBOCAN estimates of incidence and mortality worldwide for 36 cancers in 185 countries. CA Cancer J Clin.

[4] Duffy SW, Tabar L, Yen AM, Dean PB, Smith RA, Jonsson H (2020). Mammography screening reduces rates of advanced and fatal breast cancers: Results in 549,091 women. Cancer.

[5] Wolff AC, Hammond MEH, Allison KH, Harvey BE, Mangu PB, Bartlett JMS (2018). Human Epidermal Growth Factor Receptor 2 Testing in Breast Cancer American Society of Clinical Oncology/College of American Pathologists Clinical Practice Guideline Focused Update. Arch Pathol Lab Med.

[6] Harbeck N, Penault-Llorca F, Cortes J, Gnant M, Houssami N, Poortmans P (2019). Breast cancer Nat Rev Dis Primers.

[7] Pan H, Gray R, Braybrooke J, Davies C, Taylor C, McGale P (2017). 20-Year Risks of Breast-Cancer Recurrence after Stopping Endocrine Therapy at 5 Years. N Engl J Med.

[8] Fisher B, Ore L (1993). On the underutilization of breast-conserving surgery for the treatment of breast cancer. Ann Oncol.

[9] Fisher B, Slack NH, Cavanaugh PJ, Gardner B, Ravdin RG (1970). Postoperative radiotherapy in the treatment of breast cancer: results of the NSABP clinical trial. Ann Surg.

[10] Fisher B, Anderson S, Bryant J, Margolese RG, Deutsch M, Fisher ER (2002). Twenty-year follow-up of a randomized trial comparing total mastectomy, lumpectomy, and lumpectomy plus irradiation for the treatment of invasive breast cancer. N Engl J Med.

[11] Albertini JJ, Lyman GH, Cox C, Yeatman T, Balducci L, Ku N (1996). Lymphatic mapping and sentinel node biopsy in the patient with breast cancer. JAMA.

[12] Veronesi U, Paganelli G, Galimberti V, Viale G, Zurrida S, Bedoni M (1997). Sentinel-node biopsy to avoid axillary dissection in breast cancer with clinically negative lymph-nodes. Lancet.

[13] Agarwal S, Pappas L, Neumayer L, Kokeny K, Agarwal J (2014). Effect of breast conservation therapy vs mastectomy on disease-specific survival for early-stage breast cancer. JAMA Surg.

[14] Early Breast Cancer Trialists' Collaborative G (2018). Long-term outcomes for neoadjuvant versus adjuvant chemotherapy in early breast cancer: meta-analysis of individual patient data from ten randomised trials. Lancet Oncol.

[15] Sampat MB, Sirsat MV, Gangadharan P (1977). Prognostic significance of blood vessel invasion in carcinoma of the breast in women. J Surg Oncol.

[16] Lauria R, Perrone F, Carlomagno C, De Laurentiis M, Morabito A, Gallo C (1995). The prognostic value of lymphatic and blood vessel invasion in operable breast cancer. Cancer.

[17] Henderson IC, Patek AJ (1998). The relationship between prognostic and predictive factors in the management of breast cancer. Breast Cancer Res Treat.

[18] Perou CM, Sorlie T, Eisen MB, van de Rijn M, Jeffrey SS, Rees CA (2000). Molecular portraits of human breast tumours. Nature.

[19] Zheng H, Luo L, Zhao W (2020). Factors associated with level III lymph nodes positive and survival analysis of its dissection in patients with breast cancer. Laparoscopic, Endoscopic and Robotic Surgery.

[20] Houssami N, Macaskill P, Marinovich ML, Dixon JM, Irwig L, Brennan ME (2010). Meta-analysis of the impact of surgical margins on local recurrence in women with early-stage invasive breast cancer treated with breast-conserving therapy. Eur J Cancer.

[21] Pilewskie M, Morrow M (2018). Margins in breast cancer: How much is enough?. Cancer.

[22] Invernizzi M, Corti C, Lopez G, Michelotti A, Despini L, Gambini D (2018). Lymphovascular invasion and extranodal tumour extension are risk indicators of breast cancer related lymphoedema: an observational retrospective study with long-term follow-up. BMC Cancer.

[23] Britto AV, Schenka AA, Moraes-Schenka NG, Alvarenga M, Shinzato JY, Vassallo J (2009). Immunostaining with D2–40 improves evaluation of lymphovascular invasion, but may not predict sentinel lymph node status in early breast cancer. BMC Cancer.

[24] Invernizzi M, Michelotti A, Noale M, Lopez G, Runza L, Giroda M (2019). Breast Cancer Systemic Treatments and Upper Limb Lymphedema: A Risk-Assessment Platform Encompassing Tumor-Specific Pathological Features Reveals the Potential Role of Trastuzumab. J Clin Med.

[25] Dinshaw KA, Budrukkar AN, Chinoy RF, Sarin R, Badwe R, Hawaldar R (2005). Profile of prognostic factors in 1022 Indian women with early-stage breast cancer treated with breast-conserving therapy. Int J Radiat Oncol Biol Phys.

[26] Nichol AM, Chan EK, Lucas S, Smith SL, Gondara L, Speers C (2017). The Use of Hormone Therapy Alone Versus Hormone Therapy and Radiation Therapy for Breast Cancer in Elderly Women: A Population-Based Study. Int J Radiat Oncol Biol Phys.

[27] Lee BM, Chang JS, Cho YU, Park S, Park HS, Kim JY (2018). External validation of IBTR! 2.0 nomogram for prediction of ipsilateral breast tumor recurrence. Radiat Oncol J.

[28] Chen SY, Tang Y, Song YW, Wang SL, Jin J, Liu YP (2018). Prognosis and risk factors of 1 791 patients with breast cancer treated with breast-conserving surgery based on real-world data. Zhonghua Zhong Liu Za Zhi.

[29] NCCN Clinical Practice Guidelines in Oncology (NCCN Guidelines®) Breast Cancer Version 3.2021 2021. Available from: https://www.nccn.org/professionals/physician_gls/pdf/breast.pdf.

[30] Burstein HJ, Curigliano G, Loibl S, Dubsky P, Gnant M, Poortmans P (2019). Estimating the benefits of therapy for early-stage breast cancer: the St. Gallen International Consensus Guidelines for the primary therapy of early breast cancer 2019. Ann Oncol.

[31] Park YH, Senkus-Konefka E, Im SA, Pentheroudakis G, Saji S, Gupta S (2020). Pan-Asian adapted ESMO Clinical Practice Guidelines for the management of patients with early breast cancer: a KSMO-ESMO initiative endorsed by CSCO, ISMPO, JSMO, MOS. SSO and TOS Ann Oncol.

[32] Moher D, Liberati A, Tetzlaff J, Altman DG, P Group (2009). Preferred reporting items for systematic reviews and meta-analyses: the PRISMA statement. BMJ.

[33] Page MJ, McKenzie JE, Bossuyt PM, Boutron I, Hoffmann TC, Mulrow CD (2021). The PRISMA 2020 statement: an updated guideline for reporting systematic reviews. BMJ.

[34] Sterne JAC, Savovic J, Page MJ, Elbers RG, Blencowe NS, Boutron I (2019). RoB 2: a revised tool for assessing risk of bias in randomised trials. BMJ.

[35] GA Wells BS, D O'Connell, J Peterson, V Welch, M Losos, P Tugwell. The Newcastle-Ottawa Scale (NOS) for assessing the quality of nonrandomised studies in meta-analyses http://www.ohri.ca/programs/clinical_epidemiology/oxford.asp2019 [updated October 16, 2020; cited 2020 October 16]. Available from: http://www.ohri.ca/programs/clinical_epidemiology/oxford.asp.

[36] Higgins JP, Thompson SG (2002). Quantifying heterogeneity in a meta-analysis. Stat Med.

[37] Magee B, Swindell R, Harris M, Banerjee SS (1996). Prognostic factors for breast recurrence after conservative breast surgery and radiotherapy: results from a randomised trial. Radiother Oncol.

[38] Yoshida T, Takei H, Kurosumi M, Ninomiya J, Ishikawa Y, Hayashi Y (2009). Ipsilateral breast tumor relapse after breast conserving surgery in women with breast cancer. Breast.

[39] Yi OV, Lee JW, Kim HJ, Lim WS, Park EH, Lee JS (2009). Risk Factors of Local Recurrence after Breast Conserving Therapy in Invasive Breast Cancer. J Breast Canc.

[40] Lupe K, Truong PT, Alexander C, Lesperance M, Speers C, Tyldesley S (2011). Subsets of women with close or positive margins after breast-conserving surgery with high local recurrence risk despite breast plus boost radiotherapy. Int J Radiat Oncol Biol Phys.

[41] Adkins FC, Gonzalez-Angulo AM, Lei X, Hernandez-Aya LF, Mittendorf EA, Litton JK (2011). Triple-negative breast cancer is not a contraindication for breast conservation. Ann Surg Oncol.

[42] Freedman GM, Li T, Polli LV, Anderson PR, Bleicher RJ, Sigurdson E (2012). Lymphatic space invasion is not an independent predictor of outcomes in early stage breast cancer treated by breast-conserving surgery and radiation. Breast J.

[43] Mittendorf EA, Buchholz TA, Tucker SL, Meric-Bernstam F, Kuerer HM, Gonzalez-Angulo AM (2013). Impact of chemotherapy sequencing on local-regional failure risk in breast cancer patients undergoing breast-conserving therapy. Ann Surg.

[44] Perez CA, Zumsteg ZS, Gupta G, Morrow M, Arnold B, Patil SM (2013). Black race as a prognostic factor in triple-negative breast cancer patients treated with breast-conserving therapy: a large, single-institution retrospective analysis. Breast Cancer Res Treat.

[45] Matsuda N, Hayashi N, Ohde S, Yagata H, Kajiura Y, Yoshida A (2014). A nomogram for predicting locoregional recurrence in primary breast cancer patients who received breast-conserving surgery after neoadjuvant chemotherapy. J Surg Oncol.

[46] Park JS, Choi DH, Huh SJ, Park W, Kim YI, Nam SJ (2015). Comparison of Clinicopathological Features and Treatment Results between Invasive Lobular Carcinoma and Ductal Carcinoma of the Breast. J Breast Cancer.

[47] Sopik V, Nofech-Mozes S, Sun P, Narod SA (2016). The relationship between local recurrence and death in early-stage breast cancer. Breast Cancer Res Treat.

[48] Altman DG, Bland JM (2011). How to obtain the confidence interval from a P value. BMJ.

[49] Dinshaw KA, Sarin R, Budrukkar AN, Shrivastava SK, Deshpande DD, Chinoy RF (2006). Safety and feasibility of breast conserving therapy in Indian women: two decades of experience at Tata Memorial Hospital. J Surg Oncol.

[50] McCahill LE, Single RM, Aiello Bowles EJ, Feigelson HS, James TA, Barney T (2012). Variability in reexcision following breast conservation surgery. JAMA.

[51] Darby S, McGale P, Correa C, Taylor C, Arriagada R, Early Breast Cancer Trialists' Collaborative G (2011). Effect of radiotherapy after breast-conserving surgery on 10-year recurrence and 15-year breast cancer death: meta-analysis of individual patient data for 10,801 women in 17 randomised trials. Lancet.

[52] Jagsi R, Li Y, Morrow M, Janz N, Alderman A, Graff J (2015). Patient-reported Quality of Life and Satisfaction With Cosmetic Outcomes After Breast Conservation and Mastectomy With and Without Reconstruction: Results of a Survey of Breast Cancer Survivors. Ann Surg.

[53] Korde LA, Somerfield MR, Carey LA, Crews JR, Denduluri N, Hwang ES (2021). Neoadjuvant Chemotherapy, Endocrine Therapy, and Targeted Therapy for Breast Cancer: ASCO Guideline. J Clin Oncol.

[54] Hamy AS, Lam GT, Laas E, Darrigues L, Balezeau T, Guerin J (2018). Lymphovascular invasion after neoadjuvant chemotherapy is strongly associated with poor prognosis in breast carcinoma. Breast Cancer Res Treat.

