# Supplementary material for: Lympho-vascular invasion impacts the prognosis in breast-conserving surgery: a systematic review and meta-analysis
Source: BMC Cancer. 2022 Jan 25;22:102. doi: 10.1186/s12885-022-09193-0 (PMC8787911; doi:10.1186/s12885-022-09193-0)
Supplement: Supplementary file 1 — Additional file 1. [file 12885_2022_9193_MOESM1_ESM.docx]

**Supplementary**

**Search strategy:**

PubMed: ((breast conservative therapy) OR (breast conserving surgery) OR (reserved mastectomy)) AND ((lymphovascular invasion) OR (lympho-vascular invasion) OR (lympho vascular invasion) OR (tumor thrombus) OR (carcinoma embolus))

Embase: ('breast conserving surgery':ab,ti OR 'breast-conserving surgery':ab,ti OR 'breast conservative therapy':ab,ti OR 'reserved mastectomy':ab,ti) AND ('lymphovascular invasion':ab,ti OR 'lympho-vascular invasion':ab,ti OR 'lympho vascular invasion':ab,ti OR 'tumor thrombus':ab,ti OR 'carcinoma embolus':ab,ti)

Web Of Science: ((breast conserving surgery) OR (breast-conserving surgery) OR (breast conservative therapy) OR (reserved mastectomy)) AND ((lymphovascular invasion) OR (lympho-vascular invasion) OR (lympho vascular invasion) OR (tumor thrombus) OR (carcinoma embolus))

Cochrane Library: ("breast cancer"):ti,ab,kw AND ("breast conserving surgery"):ti,ab,kw AND ("lymphovascular invasion"):ti,ab,kw
